# Generalist, specialist, or expert in palliative care? A cross-sectional open survey on healthcare professionals’ self-description

**DOI:** 10.1186/s12904-024-01449-9

**Published:** 2024-05-16

**Authors:** Ingrid van Zuilekom, Suzanne Metselaar, Fleur Godrie, Bregje Onwuteaka-Philipsen, Harmieke van Os-Medendorp

**Affiliations:** 1 Saxion, University of Applied Science, School of Health, research group Smart Health, Postbus 70.000, 7500 KB Enschede, The Netherlands; 2 Amsterdam UMC Location VUmc, De Boelelaan 1117 1081 HV Amsterdam Postbus 7057, 1007 MB Amsterdam, The Netherlands; 3 Amsterdam UMC Location VUmc, Department of Ethics, Law and Humanities, De Boelelaan 1117 1081 HV Amsterdam Postbus 7057, 1007 MB Amsterdam, The Netherlands; 4 Amsterdam UMC Location VUmc, Chair Amsterdam UMC Expertise Center for Palliative Care, Department of Public and Occupational Health, Locatie VUmc | MF D349 | van der Boechorststraat 7, 1081BT Amsterdam, The Netherlands; 5 Domain of Health, Sports and Welfare, Inholland, University of Applied Sciences, De Boelelaan, 1109, 1081 HV Amsterdam, The Netherlands; 6 Spaarne Gasthuis Academy, Spaarnepoort 1, 2134 TM Hoofddorp, The Netherlands

**Keywords:** Palliative care, Specialist expertise, Self-description, Work experience, Postgraduate education

## Abstract

**Background:**

In the Netherlands, palliative care is provided by generalist healthcare professionals (HCPs) if possible and by palliative care specialists if necessary. However, it still needs to be clarified what specialist expertise entails, what specialized care consists of, and which training or work experience is needed to become a palliative care specialist. In addition to generalists and specialists, ‘experts’ in palliative care are recognized within the nursing and medical professions, but it is unclear how these three roles relate. This study aims to explore how HCPs working in palliative care describe themselves in terms of generalist, specialist, and expert and how this self-description is related to their work experience and education.

**Methods:**

A cross-sectional open online survey with both pre-structured and open-ended questions among HCPs who provide palliative care. Analyses were done using descriptive statistics and by deductive thematic coding of open-ended questions.

**Results:**

Eight hundred fifty-four HCPs filled out the survey; 74% received additional training, and 79% had more than five years of working experience in palliative care. Based on working experience, 17% describe themselves as a generalist, 34% as a specialist, and 44% as an expert. Almost three out of four HCPs attributed their level of expertise on both their education and their working experience. Self-described specialists/experts had more working experience in palliative care, often had additional training, attended to more patients with palliative care needs, and were more often physicians as compared to generalists. A deductive analysis of the open questions revealed the similarities and distinctions between the roles of a specialist and an expert. Seventy-six percent of the respondents mentioned the importance of having both specialists and experts and wished more clarity about what defines a specialist or an expert, how to become one, and when you need them. In practice, both roles were used interchangeably. Competencies for the specialist/expert role consist of consulting, leadership, and understanding the importance of collaboration.

**Conclusions:**

Although the grounds on which HCPs describe themselves as generalist, specialist, or experts differ, HCPs who describe themselves as specialists or experts mostly do so based on both their post-graduate education and their work experience. HCPs find it important to have specialists and experts in palliative care in addition to generalists and indicate more clarity about (the requirements for) these three roles is needed.

**Supplementary Information:**

The online version contains supplementary material available at 10.1186/s12904-024-01449-9.

## Background

The need for palliative care is increasing due to a growing population of individuals with dementia, cancer, or other life-threatening or incurable diseases [1–3]. Palliative care aims to improve the quality of their life and that of their families [4–6]. Palliative care is an approach to alleviating physical, psychosocial, and spiritual suffering in patients and their families facing a life-threatening illness [4]. This approach intends to integrate palliative care methods and procedures in general care settings [7]. As so many people need palliative care, it has been argued that *all* healthcare professionals (HCPs) should be able to provide it. This entails, among other things, that HCPs should be able to provide advance care planning, align treatment with a patient’s goals, wishes, and needs, and essential symptom management, avoid futile or burdensome treatments, and care for people who are dying [8–10].

In complex care situations, however, generalists may be supported by palliative care specialists. [11–13]. This support pertains to consultation on palliative care issues or the transfer of care when this is indicated [14, 15]. This collaboration between generalists and specialists is considered to be essential for the quality of care [16–18]. Brinkman et al. described that specialist palliative care has positive effects on the quality of life, and the symptom burden of patients with advanced cancer has increased over the past years [19]. Temel [20] stated that involving specialized palliative care early in the trajectory improves multiple outcomes among patients with advanced cancer and their caregivers. Boddaert et al. [21] described that early provision of specialist palliative care for patients with advanced disease of frailty strongly relates to a better quality of life, less depression and anxiety, and higher satisfaction with care.

Therefore, a so-called ‘mixed model’ of palliative care, combines primary-level palliative care (i.e., skills that all HCPs should have or ‘palliative care generalists’) with specialty-level palliative care (i.e., skills for managing more complex and challenging cases or ‘palliative care specialists’) [7, 22]. Such a mixed generalist-specialist model decreases the likelihood of potentially inappropriate end-of-life care for patients [21] and promotes overall satisfaction with care [23].

Most countries nowadays distinguish between general and specialized palliative care [24, 25] including the Netherlands. However, in the Netherlands, it is not yet clear what exactly this distinction entails. Although a competency framework for the generalist in palliative care has been established, on which there is nationwide consensus, such a framework does not yet exist for the specialist in palliative care [26]. Palliative care is not a formal specialty in the Netherlands. Although different training programs in palliative care are available, it is unclear through which training you become a palliative care specialist.

In the Netherlands Quality Framework for Palliative Care (NQFPC), broad descriptions are given of the generalist and specialist. In addition, an expert in palliative care is recognized (see Table 1). However, these descriptions are very general and do not provide sufficient clarity and precision. For instance, it is unclear which training and competencies are needed to become a specialist or expert in palliative care and how the role of a specialist relates to the role of a generalist in palliative care practice. The NQFPC also does not specify when and how specialists and generalists should collaborate. Furthermore, the NQFPC distinguishes a third level of expertise: the role of an expert [27]. However, no clear definition is given of the expert in palliative care and we neither found such a description in international literature.

**Table 1:** Description of generalist, specialist, and expert palliative care in the Netherlands Quality Framework for Palliative Care (NQFPC) [27]

NPCQF described the definition of the palliative care specialist as “The palliative care specialist is qualified through recognized training in palliative care and has specific knowledge and skills in more complex palliative care. Often the palliative care specialist, from his position working in a field where palliative care is frequently part of the daily practice but is not the main focus.
The palliative care specialist is preferably embedded in a specialized palliative care team. The specialist knows his limitations and consults a palliative care expert if necessary”. The definition of the role of the palliative care expert:
“The palliative care expert is qualified through a recognized training in palliative care, work experience, and substantive deepening and broadening and has specific knowledge and skills in complex palliative care and crises The palliative care expert works from his position in a field where palliative care is the only focus of the work. The palliative care expert is preferably embedded in a specialized team”.

This unclarity may undermine the quality of palliative care. For instance, it may delay the initiation and referral to specialized palliative care [28, 29]. In addition, as a well-defined competency profile for specialists is lacking, and it is unclear whether people can call themselves ‘specialists’ after completing a course in palliative care.

In this study, we aim to explore how HCPs working in palliative care described themselves in terms of generalist, specialist, and expert, based on the description of the Netherlands Quality Framework for Palliative Care (NQFPC) [27, 30] and how this self-description is related to work experience and education. Our secondary aim is to explore differences between the role of generalists and the combined role of specialist/ expert regarding work characteristics and to explore considerations of all HCPs regarding the distinction between palliative care specialists and experts.

## Methods

### Design and setting

A cross-sectional study was carried out using an open online survey. The survey period lasted for five months (May -September 2022). This study is embedded in the Dutch ‘Optimizing Education and Training in Palliative Care’ (O^2^PZ) program, which aims to accomplish that every HCP in the Netherlands has the right competencies to provide high-quality palliative care [31].

### Population

The target group of the survey concerns all HCPs of every Netherlands qualification framework (NLQF) level who have an affinity with and/or provide palliative care. The NLQF level is based on the European Qualifications Framework (EQF), consisting of eight levels of learning outcomes. In the Netherlands, we use the following distinctions: nurse assistants are trained at NLQF levels 2 and 3, nurse intermediate vocational levels at NLQF level 4, and bachelor nurses at NLQF level 5 or 6. Clinical nurse specialists and Physician Assistants (PA) are trained at NLQF level 7, and physicians and specialized physicians at NLQF level 8 and 8+. These levels are based on knowledge, skills, independence, and responsibilities [32].

### Recruitment

The survey was published in digital newsletters, and on websites of organizations like the Dutch Society of Professionals in Palliative Care (Palliactief) and via Linkedin. The Dutch Nurses Association (V&VN) disseminated the questionnaire among their members, including nurse assistants, nurses (bachelor and vocation level), clinical nurse specialists. HCPs affiliated with the O^2^PZ program were asked to share the questionnaire with other HCPs, especially those who provide palliative care at the bedside.

### Variables and data collection

The questionnaire (see additional file 1) consisted of 16 mostly closed but also some open-ended questions. Except for question 13, and 3 out of 5 items of question 14, all questions have been analyzed for this article. Question 13 and parts of question 14 deal with when to consult a specialist or expert, which will be discussed in another article.

The primary outcome of this study is the self-description of HCPs working in palliative care regarding their role as generalists, specialists, or experts in palliative care and how this is related to their work experience and education. We focus on the threefold generalist – specialist – expert**.** This was measured by asking questions 11 and 12 (see additional file 1).

To compare generalists with the combined role of specialists and experts, we also collected demographic and work-related characteristics, such as age, sex, current work setting, number of patients seen in the last year, and current job title (questions 1 to 7). It is expected that HCPs will not have a clear description of what they mean by specialist or expert and what distinguishes them or is similar, so we decided to combine the roles of specialist and expert (Table 5).

The considerations of HCPs working in palliative care and the distinction and similarities between the role of specialist and expert were collected using two open questions, 14 and 15.

The questionnaire was developed by five researchers based on their practical and academic experiences in palliative care (authors IZ, SM, FG, BOP, and HOM) and based on relevant peer-reviewed literature [33, 34]. In the literature, we searched for competencies that fit HCPs specialized in palliative care. Forbat et al. [9] stated that communication and listening competencies are described as typical skills for palliative care specialists. Brown et al. [14] noted that specialists should have skills for managing complex and difficult cases and co-exist to support generalists through consultation care and transfer of care. Gamondi et al. [7] described ten core interdisciplinary competencies in palliative care. Radbruch et al. [35] developed a framework that described the standards and norms for hospice and palliative care in Europe.

The content and structure of the questionnaire were presented to a working group. The authors approached potential participants for the working group using the following selection criteria: gender, affinity with palliative care, and a mix of generalists and specialists working in different healthcare domains. The working group comprised ten healthcare providers: five nurses and five physicians.

The questionnaire was adjusted based on their feedback. It was conducted using the survey tool Survalyzer provided by Amsterdam UMC.

### Analysis

Quantitative analyses were performed using IBM SPSS version 28 (IBM Corp., Armonk, NY, USA). Respondents who did not work in direct patient care were excluded. Respondents who did not describe themselves as generalist, specialist, or expert based on *education or based on work-experience* were categorized as generalists because of their initial education as HCPs. Thereby, we followed Boddaert et al. [27, 30] who described in the National Quality Framework Palliative Care: In the Netherlands, all HCPs are expected to provide generalist palliative care, informed by national standards and guidelines. Palliative care specialists can be consulted to provide support and expert advice [14, 22, 29, 36]. This was also stated by Gamondi [7] in the Core Competencies in Palliative Care by the European Association Palliative Care: “*All healthcare professionals and workers should be able to provide appropriate palliative care and thus need to be trained to provide the highest possible standards of care to meet the challenging needs of patients and families, irrespective of diagnosis”.*

Descriptive statistics were used to summarize baseline characteristics of healthcare professionals and to describe the number and percentages of self-described roles of HCPs, based on work experience and education. Hereby, we used the threefold distinction of generalist—specialist—expert, based on the context in the Netherlands and described in the Netherlands Quality Framework for Palliative Care (NQFPC) [27, 30]. A Chi-square test was used to test for significant differences between self-description based on education or work experience and other characteristics.

Secondly, we analyzed differences between generalists and the combined roles of specialist and expert. We combined the roles of specialist and expert because, in the international literature, the role of an expert was not recognized as a role next to the role of a specialist [36–39]. In Dutch palliative care practice, these roles are used interchangeably. Because of the number of tests, p values <.01 were considered statistically significant.

Questions 14 and 15 (open-ended questions regarding the distinction between specialist and expert palliative care) were analyzed deductively, based on the role description of specialist and expert in the NQFPC [27, 30] using Excel. Answers were read and re-read by the first and third authors, and the final analyses were discussed with all authors.

### Ethics

All respondents were carefully informed about the study objectives and were asked for informed consent, which was the survey’s first question. An email with information about consent and confidentiality of the survey data was sent. The email also included a link to the online questionnaire. The question for consent was the first question of the online questionnaire, which stated: ‘Do you consent to use your answers to the questions in the questionnaire for research purposes?’

If this question was answered positively, the respondents could continue the questionnaire; if they did not give permission, they could not continue the questionnaire. Informed consent was obtained from all the respondents. All data were collected and analyzed anonymously. We performed our study in accordance with the relevant guidelines and regulations of the Declaration of Helsinki. According to the Medical Research Involving Human Subjects Act, our study is exempted by formal review by an ethics committee [40, 41]. Participants were not asked to act or to change behaviors, and the questions were not of a drastic nature. According to Dutch law (WMO) [40] no permission is required from an Ethic Committee [41], only a self-check of the Saxion Ethics Advise Ethic Committee (SEAC).

## Results

### Respondent characteristics

The online survey was accessed 1002 times, and 854 participants consented to use their answers/data and filled out the questionnaire. The final number of participants differed per question. Of the participants, 87% were female, 55% were older than 51 years, 28% worked in home care, 21% worked in a hospital and almost 18% worked in a hospice setting (Table 2). Most participants had a profession as a bachelor or vocational trained nurse or physician. 32% of respondents had more than 20 years of work experience in palliative care. 33% of the respondents provided palliative care to more than 60 patients annually. 74% of the respondents followed additional training for palliative care.

**Table 2:** Summary of respondent characteristics (*N*=854)

**Respondent characteristics**	
**Age in years**	
< 30	61 (7.2%)
31-40	107 (12.6%)
41-50	213 (25.1%)
51-60	321 (37.9%)
> 60	145 (17.1%)
**Gender**	
Female	743 (87.1%)
Male	107 (12.5%)
Other	3 (0.4%)
**Work setting**	
Home care	233 (28.0%)
Hospital	171 (20.5%)
Hospice	149 (17.9%)
Nursing home	124 (14.9%)
General practitioner	69 (8.3%)
Care for disabled persons	22 (2.6%)
Setting unknown	19 (2.3%)
Education	16 (1.9%)
Consortium	12 (1.4%)
Psychiatry	9 (1.1%)
Research	3 (0.4%)
Other^a^	6 (0.7%)
**Function**	
Bachelor nurse	235 (28.7%)
Physicians	189 (23.0%)
Nurse (vocational level)	182 (22.2%)
Clinical nurse specialist/PA	72 (8.8%)
Nurse Assistant	70 (8.5%)
Not working in direct patient care:	
Manager	15 (1.8%)
Coordinator Consortium	9 (1.1%)
Education	8 (1.0%)
Volunteer	6 (0.7%)
Chaplain	5 (0.6%)
Researcher	3 (0.4%)
Other	26 (3.2%)
**Work experience in years**	
0-5	50 (6.2%)
6-10	79 (9.8%)
11-20	170 (21.0%)
>20	511 (63.1%)
**Work experience in palliative care over the years**	
Not providing palliative care	51 (6.3%)
0-5	123 (15.2%)
6-10	173 (21.4%)
11-20	201 (24.8%)
>20	262 (32.3%)
**Number of patients with palliative needs to be seen in the last year**	
** Not providing palliative care**	72 (9.0%)
1-10	127 (15.9%)
11-20	115 (14.4%)
21-40	131 (16.4%)
41-60	87 (10.9%)
>60	268 (33.5%)
**Followed an additional training in palliative care (*N*=785)**	
No	147 (26.0%)
Yes, for physicians	180 (31.8%)
Yes, for nurses	153 (27.0%)
Yes, for clinical nurse specialists/PA^b^	45 (8.0%)
Yes, for nurse assistants	41 (7.2%)
**Which additional training courses (*N*=489)**	
Postgraduate nurses^c^	226 (46.2%)
Postgraduate physicians	118 (24.1%)
Basic course	37 (7.6%)
Course physicians	21 (4.3%)
Other	87 (17.8%)

^a^Other: police officers, retired nurses

^b^/PA/Physician Assistant

^c^Postgraduate courses nurses: > 21 days, introductory course PC 3 – 3 -7 days, postgraduate courses physicians (incl Cardiff) > 20 days and course nine days. Other: E-learning, symposia, webinar

Based on the descriptions of generalist, specialist, and expert palliative care in the Netherlands Quality Framework for Palliative Care (NQFPC, Table 1), most respondents described themselves as either a specialist or an expert: *based on education*, this was respectively 31% and 38%. The number of participants who described themselves as specialist or expert, *based on work experience*, was respectively 34% and 44% (Table 3). 17% of the respondents described themselves as a generalist based on work experience and 25% described themselves as a generalist based on education.

**Table 3:** Self-description by healthcare professionals of their role, based on work experience and based on education

**Self-description**^**b**^	**No description**	**Generalist**	**Specialist**	**Expert**	***p*<0.001**
**Self-description based on education**^**a**^	36 (5.7%)	159 (25.3%)	193 (30.7%)	240 (38.2%)	
**Self-description based on work experience**^**a**^	29 (4.6%)	109 (17.4%)	211 (33.8%)	279 (44.4%)	

^a^Percentage in row

^b^Only healthcare professionals are included in the analysis

Table 4 shows a significant difference in self-description when self-description is based on education or work experience (p<0.001). 23% of the participants gave a different self-description in terms of generalist, specialist, or expert in palliative care if the basis was education or work experience; 70% of the participants gave the same self-description when this was based on education or work experience.

**Table 4:** Comparison of self-description of work experience and education^a^

		**Self- description based on work experience**	**No description**	**Generalist**	**Specialist**	**Expert**	**Total self-description based on work experience**	*P*<0.001
**Self-description based on education**	**No description**		48 (7.1%)	4 (0.6%)	4 (0,6%)	7 (1.0%)	63	
	**Generalist**		2 (0.3%)	106 (15.8%)	40 (5.9%)	21 (3.1%)	169	
	**Specialist**		2 (0.3%)	7 (1.0%)	145 (21.5%)	41 (6.1%)	195	
	**Expert**		1 (0.1%)	2 (0.3%)	26 (3.9%)	217 (32.2%)	246	
		*Total self-description based on education*	53	119	215	286		

^a^Percentage in total

Table 5 shows the differences and similarities between self-described generalists and specialists/experts. It also distinguishes self-description based on education from self-description based on work experience.

**Table 5:** Background characteristics of self-described generalists in palliative care versus self-described specialists or experts in palliative care^a^

Self-description based on education				Self-description based on work experience		
	Generalist	Specialist/ Expert	Chi 2 (*p*)	Generalist	Specialist/ Expert	Chi 2 (*p*)
**Function**			<0.001			<0.001
Nurse assistant	29 (19.0%)	11 (2.6%)		24 (23.1%)	16 (3.4%)	
Nurse vocation level	46 (30.1%)	99 (23.3%)		29 (27.9%)	116 (24.5%)	
Bachelor nurse	37 (24.2%)	138 (32.5%)		28 (26.9%)	147 (31.0%)	
Clinical nurse specialists/PA^b^	15 (9.8%)	39 (9.2%)		6 (5.8%)	48 (10.1%)	
Physicians	26 (17.0%)	138 (32.5%)		17 (16.3%)	147 (31.0%)	
**Work setting**			0.068			<0.007
Hospital	32 (21.3%)	99 (23.5%)		20 (19.6%)	111 (23.7%)	
Nursing home	33 (22.0%)	48 (11.4%)		25 (24.5%)	56 (11.9%)	
Home care	43 (28.7%)	124 (29.5%)		33 (32.4%)	134 (28.6%)	
General practitioner care	14 (9.3%)	47 (11.2%)		12 (11.8%)	49 (10.4%)	
Hospice	25 (16.7%)	86 (20.4%)		10 (9.8%)	101 (21.5%)	
Psychiatry	1 (0.7%)	3 (0.7%)		0 (0.0%)	4 (0.9%)	
Care for disabled persons	2 (1.3%)	14 (3.3%)		2 (2.0%)	14 (3.0%)	
**Work experience in years**			<0.001			<0.001
0-5	24 (15.7%)	5 (1.2%)		23 (22.1%)	6 (1.3%)	
6-10	22 (14.4%)	29 (6.8%)		15 (14.4%)	36 (7.6%)	
11-20	40 (26.1%)	88 (20.7%)		21 (20.2%)	107 (22.6%)	
>20	67 (43.8%)	303 (71.3%)		45 (43.3%)	325 (68.6%)	
**Work experience providing palliative care for years**			<0.001			<0.001
0 – 5	50 (32.7%)	32 (7.5%)		40 (38.5%)	42 (8.9%)	
6 – 10	38 (24.8%)	91 (21.4%)		18 (17.3%)	111 (23.4%)	
11 – 20	33 (21.6%)	133 (31.3%)		26 (25.0%)	140 (29.5%)	
>20	32 (20.9%)	169 (39.8%)		20 (19.2%)	181 (38.2%)	
**Number of patients with palliative needs seen last yea**			<0.001			<0.001
1-10	43 (28.7%)	34 (8.1%)		38 (37.3%)	39 (8.3%)	
11-20	39 (26.0%)	46 (10.9%)		26 (25.5%)	59 (12.6%)	
21-40	20 (13.3%)	85 (20.2%)		15 (14.7%)	90 (19.2%)	
41-60	18 (12.0%)	55 (13.1%)		12 (11.8%)	61 (13.0%)	
>60	30 (20.0%)	201 (47.7%)		11 (10.8%)	220 (46.9%)	
**Additional training in palliative care**			<0.001			<0.001
No additional training	53 (36.3%)	7 (2.8%)		36 (37.5%)	24 (8.1%)	
Yes, for nurse assistants	16 (11.0%)	11 (4.5%)		11 (11.5%)	16 (5.4%)	
Yes, for nurses	50 (34.2%)	63 (25.6%)		32 (33.3%)	81 (27.4%)	
Yes, for clinical nurse specialists /PA	6 (4.1%)	27 (11.0%)		1 (1.0%)	32 (10.8%)	
Yes, for physicians	21 (14.4%)	138 (56.1%)		16 (16.7%)	143 (48.3%)	
**Type of additional training courses**^**c**^			<0.001			<0.001
Introductory course	15 (25.9%)	12 (3.4%)		9 (22.5%)	18 (4.8%)	
Course physicians	4 (6.9%)	14 (3.9%)		2 (5.0%)	16 (4.3%)	
Postgraduate nurses	7 (12.1%)	184 (51.4%)		8 (20.0%)	183 (48.7%)	
Postgraduate physicians	7 (9.6%)	101 (28.2%)		6 (15.0%)	102 (27.1%)	
Other	25 (43.1%)	47 (13.1%)		15 (37.5%)	57 (15.2%)	

^a^Percentage in column

^b^PA Physician Assistant

^c^Postgraduate courses nurses: > 21 days, introductory course 3 – 3 -7 days, postgraduate courses physicians (incl Cardiff) > 20 days and course nine days. Other: E-learning, symposia, webinar

Generalists were often nurse assistants or nurses on a vocational level, while specialists and experts were more often Bachelor Nurses or physicians.

Generalists and specialists/experts all worked in different healthcare settings. Generalists worked more often in nursing homes than specialists/experts, who mostly worked in hospitals and hospices. In other work settings, only minor differences were seen between percentages of generalists versus specialists/experts. Specialists/experts had significantly more years of work experience in general care and palliative care than generalists. This counts for specialists/experts based on education and specialists/experts based on work experience.

Specialists/experts saw more patients with a palliative need annually than generalists; this counted for both specialists/experts based on education and for specialists/experts based on work experience. More than 90% of the specialists/experts followed additional training, as compared to 64% of the self-described generalists. Specialists/experts mostly followed a postgraduate education: nurses (51%) and physicians (28%). Of the respondents who received the same training, some described themselves as generalists, whereas others described themselves as specialists/experts.

#### The similarities and differences between specialists and experts in palliative care

Respondents were asked whether they find it important to have specialists and experts in palliative care (question 14). 76% of all respondents answered that having specialists and experts in palliative care is important. There was no difference in answers between respondents describing themselves as generalists (76%) or specialists/experts (76%) based on education and respondents describing themselves as generalists (76%) or specialists/experts (75.5%) based on work experience. They were asked to provide further explanation by means of an open question (question 15). The analyses revealed two themes: teamwork and differentiation. Codes by the theme of teamwork were 1) consultation and 2) the importance of collaboration. Codes by the theme differentiation were 1) distinction in required competencies, 2) importance of having both, 3) the need for clarififying the mixed model palliative care, and 4) leadership as specialist.

#### Teamwork

Respondents described the importance of collaboration and consultation between generalists, specialists, and experts to the quality of palliative care: *“Through good cooperation with specialists and experts, palliative care will increase in quality. Especially when it becomes a recognized position within the care provision”* (Clinical nurse specialist, self-description: specialist).*“Good cooperation is very important between generalist and specialist and between specialist and expert".*

Respondents indicate that they think it is important for generalists to know what they can consult specialists/experts about. *“They must consult each other timely, be aware of their boundaries, and know their limits when someone else needs to be called in”* (Bachelor Nurse, self-description: specialist).

#### Differentiation

Respondents were asked to further explain their answers about the distinctive elements of the role of specialist and expert (question 15). Many respondents maintain there is an overlap in the competencies and roles of specialist and expert, such as collaboration and consultation. Respondents distinguished specialists from experts by mentioning that the expert has outstanding competencies in a specific area of palliative care, is fully committed to palliative care, and has a leading role in consultation, research, and in palliative care initiatives. *“For me, a palliative care expert is someone who, in addition to providing good palliative care, also shows responsibility in optimizing palliative care at the meso and macro level. Examples are developing transmural care pathways, setting up training courses for nurses to become specialists, initiating quality projects, and participating in projects. The expert has a consultation function, access to a multidisciplinary network, and can work in an interprofessional team”* (Physician, self-description: specialist). *”I think the cooperation between the specialist, who is executive, and the expert who coordinates and advises is important”* (Physician, self-description: generalist).

Some respondents, however, argue that specialists also have a leading role, whereas experts distinguish themselves by fully focusing in palliative care: “*Specialists mainly have a leading role in organizing palliative care in the department, and the surplus of experts is that they have palliative care as their core business”* (Physician, self-description: expert). Finally, some respondents indicated that experts do not necessarily have patient contact, whereas others think they do.

Furthermore, respondents considered long-term work experience to be a requirement of being an expert. For instance, as a nurse argued: “*Not only by education but also by work experience you make the step from specialist to expert. You become an expert not only through theoretical knowledge but also through work experience”* (Bachelor Nurse, self-description: expert).

Yet, respondents (including respondents that answered yes to the statement that the distinction between specialist and expert is important) also considered the distinction between the role of specialist and expert as ambiguous and arbitrary and even doubted the existence of this distinction: *“In my opinion, the difference between a specialist and an expert is difficult to distinguish, and the question is whether it is necessary. What is paramount is the cooperation between specialists and experts in executive, consulting, and continuing tasks”*(Vocational level, self-description: expert). *“I still find it difficult to use this dichotomy. Especially because I have a specialist education and sufficient experience (5 years) and am involved in both medium and highly complex situations, but I work part-time as a district nurse and part-time as an expert in palliative care. How should I describe myself?”* (Bachelor Nurse, self-description: no description*).*“*I think it is a very arbitrary distinction. In practice, we do not use the terms that way either. You have a generalist, and you have a specialist. It would be best if you did not also want to add an expert in the field of work”* (Physician, self-description: specialist).

#### Need for clarification

Some respondents argued for the necessity of additional training to become a specialist or an expert in palliative care. They stressed that it should be very clear which competencies are trained, and for which role you are being trained. As one respondent noted: *“Now it is easy to say that someone specializes in palliative care, but what education or training did they receive for that? Sometimes this is said after a few hours of training* ” (Bachelor Nurse, self-description: generalist).

Respondents also described that education level plays a role in becoming a specialist or expert; they mentioned that only HCPs with higher graduate education, such as bachelor nurses, clinical nurse specialists, and physicians, can become specialists or experts. “*It is a difficult distinction between specialist and expert. Maybe the level of education in general? Only clinical nurse specialists and physicians can become specialists or experts* ”(Physician, self-description: specialist).

The importance of clarity and recognition of educational programs is stressed:* “The palliative care courses [should be] legally recognized with an official title”* (Bachelor Nurse, describes himself as a specialist), and: “*There are so many different courses in palliative care. Create an unambiguous curriculum”* (Bachelor Nurse, describes himself as a specialist). *“Which (accredited) education and work experience you must have to describe yourself as a specialist or expert is, in my opinion, not determined; it helps if we determine this”* (Physician, self-description: specialist).

Finally, respondents stressed the importance of an unambiguous profile for specialists and experts, which is recognized nationally: *“Establish a national professional profile for specialists and palliative care experts.”*

## Discussion

### Main findings

Our findings show that most respondents described themselves as specialists or experts in the Dutch mixed model palliative care and less often as generalists. Three out of four HCPs indicated having the same level of expertise based on training as based on working experience.

Characteristics of self-described specialists/ experts significantly differ from the characteristics of self-described generalists. Self-described specialists/experts are more often bachelor nurses, clinical nurse specialists, and physicians; generalists are more often nurse assistants and vocational-level nurses. Specialists/ experts more often followed additional or postgraduate education than generalists. They had more years of work experience in palliative care than generalists. Those who describe themselves as specialists/experts also care for more patients with incurable conditions or high vulnerability than generalists on an annual basis.

Almost all respondents (76%) find it important to have palliative care specialists in addition to generalists. However, respondents indicate the need for a clear profile of specialists and possibly experts, which is recognized nationally. Furthermore, the difference between a specialist and an expert is not clear to many respondents, some of whom even doubt the need for the role of expert in addition to the specialist.

### Interpretation of findings

Our results show that HCPs describes themselves as generalist, specialists, and experts on divergent grounds. Although 76% of respondents self-describe themselves on the same level of expertise based on training as based on working experience, there is a group that self-describes themselves differently based on education and based on work experience. Above that the comparison of background characteristics of self-described generalists and specialists/experts does show a tendency towards more palliative education and work experience for specialists/experts, but does also show diversity in self-description. There are for instance respondents that followed post-graduate education that consider themselves a generalist while others consider themselves a specialist or expert.

The question is what causes this diversity. The first reason is that post-graduate education in palliative care is relatively new in the Netherlands. Since the beginning of this century, there have been training courses for nurses and physicians who want to specialize in palliative care, for nurse assistants these courses have not (yet) been developed. Many respondents in this study already had a long time of work experience in palliative care and it could be possible that not everyone has had the opportunity to follow additional training on palliative care. This might explain the importance of work experience for the role of specialist or expert in palliative care.

A second possible explanation for the diversity in self-description can be caused by the premise that every HCP must be able to provide palliative care in the Netherlands [27].

Therefore, every HCP, nurse as well as physician is a generalist based on his graduate education. Many generalists work in nursing homes, where a lot of palliative care is provided. Generalists have received less additional training in palliative care, but because there are few palliative care specialists or experts working in their immediate environment, generalists may unknowingly provide specialist palliative care [21]. It is likely that this can cause role confusion and this affects the self-description. In addition, it can lead to late referrals to recognized specialists in palliative care or consultation teams, which compromises the quality of care [14, 24, 25].

A third possible explanation is that many HCPs have indicated that the distinction between specialist and expert is unclear. This is in line with the ambiguity found in the literature. The NQFPC [27] describes a threefold division: generalist- specialist- expert, but in literature, the dichotomy generalist - specialist palliative care is mainly described as mixed model palliative care [42–45]. Due to the different views on the distinction between specialist and expert, there is confusion about professional identity and self-description.

The fourth explanation is that our results also show that respondents with the same completed education in palliative care describe themselves as generalist, specialist, and expert. Hence, it needs to be clarified which education leads to the role of specialist or expert and which education is meant for generalists. In the Netherlands, a framework was recently established for generalist education in palliative care on different educational levels [26]. However, such a recognized framework is lacking for specialized palliative care, while examples are available from international literature. Autelitano et al. (2021) described a first draft of the competencies of the specialist palliative care nurses (only in hospitals) in Italy, based on the white paper on palliative care training of the European Association for Palliative Care (EAPC) [7, 46–48]. This can only be established if there is consensus regarding the role and competencies of specialist palliative care professionals.

Finally, the fifth explanation possibly relates to the special mixed model in the Netherlands, the threefold generalist – specialist – expert. In the open answers, there appears to be a lot of confusion about the roles of specialist and expert, and both differences and similarities are described. No literature has been found on the role of expert and the possible surplus of this role. Similarities have been found in respondents' beliefs regarding the roles and competencies of the specialist and expert, such as consultation and collaboration; these are aligned with the core competencies in palliative care described in the white paper of EAPC [7], such as consultation and leadership roles.

A Delphi study is recommended to reach a consensus on role description, competencies, and criteria as to which education you become a specialist or an expert and how we can define relevant work experience [49–51]. A recognized educational framework could also clarify the titles and roles of palliative care in healthcare organizations.

### Strengths and limitations

A strength of this study is that we collected data from HCPs of all educational (NLQF) levels and from all care settings in which palliative care is provided. This provides insight into how a broad range of HCPs that provide palliative care describe themselves, and on what basis. Some limitations should also be mentioned. Although we used different avenues to recruit HCPs, selection bias could have occurred: respondents with an affinity with palliative care may have been overrepresented. This is indicated by the fact that our respondents mainly call themselves specialists or experts. Furthermore, we included relatively few respondents who were nurse assistants or nurses at the vocational level. In reality, this is a relatively large group within healthcare and provides a major part of palliative care at the bedside. In addition, HCPs who participated in this study were most often at an advanced age and had a long work experience in palliative care. We expect this to have influenced the outcomes, for instance on the importance of work experience for becoming a specialist.

## Conclusions

Among HCPs working in palliative care in the Netherlands, 17 % describe themselves as a generalist, 34% as a specialist, and 44% as an expert based on work experience. Almost three out of four HCPs gave the same self-description when this was based on their education and their working experience.

We showed considerable differences between generalists versus specialists/experts regarding function, work experience (in palliative care), the number of patients seen annually, and postgraduate education. Participants also mentioned similarities between specialists and experts in being consulted and the importance of collaborating and having a leadership role. HCPs (76%) find it important to have specialists and experts in palliative care in addition to generalists. Still, the Dutch participants need more clarity about the distinction between both roles and with which education and work experience you become a specialist or an expert. We, therefore, recommend reaching a consensus about the role description and competencies of specialists and experts and how these roles relate to general palliative care, including educational requirements and relevant work experience.

### Supplementary Information

Supplementary Material 1.

## Data Availability

The datasets used and/or analyzed during the current study are available from the corresponding author on reasonable request.

## Abbreviations

HCPs: Healthcare professionals
NQFPC: Netherlands Quality Framework for Palliative Care
O^2^PZ: Optimizing Education and Training in Palliative Care
NLQF: Netherlands Qualification Framework
EQF: European Qualification Framework
PA: Physician Assistant
PC: Palliative Care
V&VN: Dutch Nurses Association
