# Supplementary material for: Generalist, specialist, or expert in palliative care? A cross-sectional open survey on healthcare professionals’ self-description
Source: BMC Palliat Care. 2024 May 16;23:120. doi: 10.1186/s12904-024-01449-9 (PMC11097520; doi:10.1186/s12904-024-01449-9)
Supplement: Supplementary file 1 — Supplementary Material 1. [file 12904_2024_1449_MOESM1_ESM.docx]

**Additional file 1 Questionnaire specialist expertise in palliative care**

**Introduction**

What happens to your data? To protect your privacy, your data will not be stored. We only keep your answers to the questions in the questionnaire. We guarantee that these answers cannot be traced back to you.

Do you consent to use your answers to the questions in the questionnaire for research purposes?

- Yes, continue
- No, You cannot complete the questionnaire without your consent

**1. What is your age? (1 answer possible)**

1. ≤ 30
2. 31-40
3. 41-50
4. 51 - 60
5. More than 61

**2. What is your gender? (1 answer possible)**

1. Man
2. Wife
3. Otherwise

**3. In which setting are you *mainly* working?**

1. Hospital
   1. Academic hospital specialism /department:
   2. General hospital specialty/department:
   3. Top clinical hospital specialty/department:
2. Nursing home
   1. Specialism/Department (open question)
3. Home care
4. General practitioners setting
5. Hospice
6. Mental health care
   1. IntramuralSpecialism:
   2. Ambulatory/Specialism:
7. Organization for persons with intellectual disability
   1. Intramural/Specialism:
   2. Ambulatory/Specialism:
8. Other, namely:

**4. You are:**

1. Caring (NLQF level 2)
2. Nurse assistant (NLQF level 3)
3. Vocational level nurse (NLQF level 4)
   1. If trained in-service: option A, B, or Z nurse
   2. I also followed the following nursing further training:
4. Bachelor Nurse (NLQF-level 6)
   1. I also followed the following nursing further training:
5. Clinical Nurse Specialist (NLQF level 7)
   1. I have the following outflow profile:

6. Physician assistant (NLQF level 7)

- 1. I have the following outflow profile:

7. Physician (NLQF Level 8)

8. Medical specialist (NLQF level 8+)

- 1. My specialism is:

9. If you do not work in direct patient care, please describe your function here:

**5. How many years of professional work experience in healthcare do you have? (1 answer possible)**

1. 0 - 5 years
2. 6 - 10 years
3. 11 - 20 years
4. More than 20 years

**6. How long do you (also) provide palliative care *(By palliative care, we mean care for people with a life-threatening condition or vulnerability, where the care is mainly focused on quality of life).***

1. I do not provide palliative care
2. 0 - 5 years)
3. 6 - 10 years
4. 11 - 20 years
5. More than 20 years

**7. How many patients with palliative care needs have you cared for in the past 12 months?**

1. I do not provide palliative care
2. 1 – 10 patients
3. 11 - 20 patients
4. 21 - 40 patients
5. 41 - 60 patients
6. More than 60 patients

**8. Have you completed additional training or training on palliative care?**

1. No
2. Yes, for nurse assistants
   1. Introductory course
   2. Otherwise:
3. Yes, for vocational level nurses
   1. Introductory course
   2. Postgraduate vocational level course
   3. Otherwise:
4. Yes, for Bachelor nurses
   1. Introductory course
   2. Postgraduate bachelor course
   3. Otherwise:
5. Yes, for clinical nurse specialists/physician assistants:
   1. Introductory course
   2. Otherwise:
6. Yes, for specialists/general practitioners:
   1. Introductory course
   2. 9-days course for specialists
   3. Postgraduate course
   4. Otherwise:

**9. The highest level of palliative care I have completed is (open answer)**

**10. Related to palliative care, I describe myself as:**

- 1. Nurse palliative care
  2. Palliative care focus center
  3. Palliative care consultant
  4. Palliative care executive physician
  5. Otherwise:

Below are several questions based on the description generalist – specialist - expert in the DPCQF (19)

Please read these descriptions before answering questions **11 till 14.**

| ***Each* caregiver is at least a generalist in palliative care and:**   1. Has basic knowledge and skills in palliative care 2. Can treat symptoms medicinally and/or non-medicamentously 3. Can communicate effectively with patients, loved ones, and other healthcare providers when it comes to    1. palliative phase    2. proactive care planning    3. joint decision-making 4. Recognizes one's limitations and knows when to consult a specialist palliative care. | **The specialist palliative care:**   1. Has followed a recognized training in palliative care 2. Can provide more complex palliative care 3. Regularly has to deal with palliative care in daily practice (but is not the core of the work) 4. Preferably part of a specialized palliative care team 5. Recognizes one's limitations and knows when to consult an expert palliative care. | **The expert palliative care:**   1. Combines a recognized education, work experience, and substantive deepening and broadening 2. Can provide complex palliative care, even in crises 3. Focus is on palliative care in daily work (the core of the work or core business) 4. Preferably part of a specialized palliative care team |
| --- | --- | --- |

**11. Based on my education(s) or training(s) in palliative care, the most appropriate description for me is the role of:**

1. Generalist
2. Specialist
3. Expert
4. None of the above

**12. Based on my work experience in palliative care, the most appropriate description for me is the role of:**

1. Generalist
2. Specialist
3. Expert
4. None of the above

**13. Have you ever consulted a specialist or expert in palliative care?**

- 1. Yes, proceed to questions 13 a, b, and c
  2. No, continue to question 14

**13. a Have you consulted a specialist or an expert palliative care in the following situations?**

|  | Often | Sometimes | Never |
| --- | --- | --- | --- |
| Signaling/marking the palliative phase |  |  |  |
| Proactive care planning |  |  |  |
| Problems in the sense of meaning domain |  |  |  |
| Problems in the social domain |  |  |  |
| Problems in the psychological domain |  |  |  |
| Problems in the physical domain |  |  |  |
| Treating symptoms |  |  |  |
| Care in the dying phase |  |  |  |
| Providing aftercare |  |  |  |
| Otherwise: |  |  |  |

**13. b Can you give an example of a complex situation where you consulted a specialist or an expert palliative care ? (Open answer)**

**13. c What is the specialist or expert palliative care position that you consult?
(if you have consulted several people, choose the position of the one you consulted the most)**

1. Caregiver (NLQF level 2)
2. Nurse assistant (NLQF level 3)
3. Vocational level nurse (NLQF level 4)
4. Bachelor Nurse (NLQF level 6)
5. Clinical Nurse Specialist (NLQF level 7)
6. Physician Assistant (NLQF level 7)
7. Physician (NLQF level 8)
8. Medical specialist (NLQF level 8+)
9. Otherwise:

**14. The following are some statements about the distinction between specialists and experts in palliative care. Indicate to what extent you agree**

|  | Strongly disagree | Disagree | Undecided | Agree | Strongly agree |
| --- | --- | --- | --- | --- | --- |
| I think it is important that there are both specialists and experts in palliative care. |  |  |  |  |  |
| It is clear when I can best consult a specialist in palliative care. |  |  |  |  |  |
| It is clear when I can best consult an expert in palliative care. |  |  |  |  |  |
| Specialists in palliative care should only consult experts in palliative care. |  |  |  |  |  |
| I don't think it's necessary to distinguish between specialists and experts in palliative care. |  |  |  |  |  |

**15. Would you like to explain your answers regarding the distinction between specialists and experts in palliative care ? Then you can describe this here:**

**16. Finally, is there anything else you want to give us?**

(For example, what helps clarify the specialist’s expertise in palliative care?).
